# “Multidisciplinary management of post- infective osteoarthritis and secondary condylar resorption of temporomandibular joint: a case report in a 9 years-old female patient and a review of literature”

**DOI:** 10.1186/s13052-022-01255-0

**Published:** 2022-05-03

**Authors:** Paola Festa, Elena Arezzo, Giulia Vallogini, Anna Chiara Vittucci, Domenico Barbuti, Angela Galeotti

**Affiliations:** 1 Dentistry Unit, Department of Pediatric Surgery, Bambino Gesù Children’s Hospital, IRCCS, Viale Ferdinando Baldelli 41, 00146 Rome, Italy; 2 Pediatric and Infectious Disease Unit, Bambino Gesù Children’s Hospital, IRCCS, Rome, Italy; 3 Radiology and Diagnostic Imaging Unit, Bambino Gesù Children’s Hospital, IRCCS, Rome, Italy

**Keywords:** Case report, Child, Osteoarthritis, Temporomandibular disorders, Temporomandibular joint

## Abstract

**Background:**

Osteoarthritis and condylar resorption of temporomandibular joint (TMJ) has rarely been reported in children as consequence of otologic disease. We describe the management of a case in a 9-year-old female as long-term complication of an otomastoiditis and review the literature currently available on this topic.

**Case presentation:**

A nine-years-old female patient referred to Emergency Room of Bambino Gesù Children’s Research Hospital, IRCCS (Rome,Italy) for an acute pain in the left preauricular area and reduced mandibular movements. In the medical history an otomastoiditis and periorbital cellulitis was reported at the age of six with complete remission of symptoms after antibiotic treatment. No recent history of facial trauma and no previous orthodontic treatment were reported. She was referred to a pediatric dentist that conducted a clinical examination according to the Diagnostic Criteria of Temporomandibular Disorders (DC/TMD) and was diagnosed with bilateral myalgia of the masticatory muscles and arthralgia at the level of the left TMJ. Then, a complete diagnostic path was performed that included multidisciplinary examinations by a rheumatologist, infectious disease specialist, ear nose and throat (ENT) doctor, a maxillofacial surgeon and a medical imaging specialist. Differential diagnosis included juvenile idiopathic arthritis, idiopathic condylar resorption, trauma, degenerative joint disease, neurological disease. Finally, unilateral post-infective osteoarthritis of the left TMJ with resorption of mandibular condyle was diagnosed. The patient went through a pharmacological therapy with paracetamol associated to counselling, jaw exercises and occlusal bite plate. After 1 month, the patient showed significant reduction of orofacial pain and functional recovery that was confirmed also one-year post-treatment. The novelty of this clinical case lies in the accurate description of the multidisciplinary approach with clinical examination, the differential diagnosis process and the management of TMD with conservative treatment in a growing patient.

**Conclusions:**

Septic arthritis of temporomandibular joint and condylar resorption were described as complications of acute otitis media and/or otomastoiditis in children. We evidenced the importance of long-term follow-up in children with acute media otitis or otomastoiditis due to the onset of TMJ diseases. Furthermore, in the multidisciplinary management of orofacial pain the role of pediatric dentist is crucial for the diagnostic and therapeutic pathway to avoid serious impairment of mandibular function.

## Background

Post-infective osteoarthritis is a rare finding in the temporomandibular joint (TMJ) where the infection can spread via the haematogenous route or can be transmitted by contiguity [1, 2]. Septic osteoarthritis of the TMJ is a rare complication of acute otitis media and otomastoiditis in children [3–6]. Clinical presentation of septic osteoarthritis of the temporomandibular joint varies from one individual to another, it may include trismus, torcicollis, impaired function of the mandible, fever, pre-auricular swelling, pain [3]. Trismus, condylar resorption, pain and impaired function are common findings in other inflammatory conditions of the temporomandibular joints such as juvenile idiopathic arthritis, idiopathic condylar resorption, chronic mandibular osteomyelitis and other degenerative joint diseases [7–11]. No previous study reported clinical examination according to the DC/TMD in children affected by post-infective osteoarthritis in the TMJ area. Differential diagnosis may be difficult to perform and addressing pain and function impairment is the main goal. A correct diagnosis is crucial for accurate and effective treatments [7, 10].

In this article, we present the management of a 9 years-old female patient with orofacial pain and impaired mandibular function due to secondary mandibular condylar resorption as a complication of an otomastoiditis. We also reviewed literature of otogenic cases of TMJ septic arthritis in children, published in English since 2000, in order to discuss long-term consequences and management strategies for the improvement of mandibular function.

Search Strategy.

To identify relevant studies from 2000 until today in English language, we systematically searched MEDLINE/PubMed, EMBASE, Cochrane Central Register of Controlled Trials.

The search strategy was carried out without language restrictions until 31st August 2021. In PubMed, the following search strategy was used: ((“Temporomandibular Joint”[Mesh]) AND “Arthritis, Infectious”[Mesh]); (“Temporomandibular Joint Disorders”[Mesh]) AND “Arthritis, Infectious”[Mesh]; (“Temporomandibular Joint Disorders”[Mesh]) and septic arthritis (as single words).

In EMABSE, the following search strategy was used: ((Temporomandibular Joint Disorders OR Temporomandibular) AND (Arthritis, Infectious OR septic arthritis).

In Cochrane the following search strategy was used: Temporomandibular Joint Disorders and septic arthritis. All the works whose pathology was not of otological origin were excluded from the research.

The keywords used were: child, osteoarthritis, temporomandibular disorders and temporomandibular joint.

## Case presentation

A nine-years-old female patient referred to the Emergency Room of Bambino Gesù Children’s Research Hospital, IRCCS (Rome, Italy) for an acute pain in preauricular area and severe impairment of mandibular function.

**Fig. 1:**
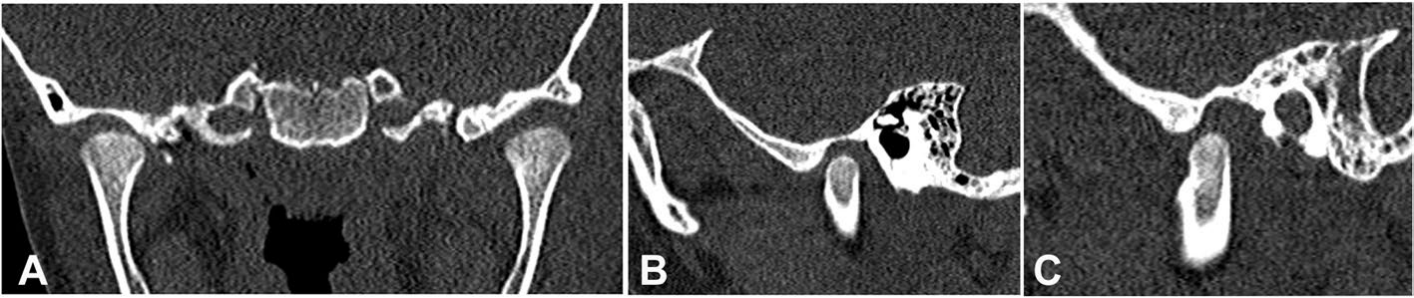
Images of maxillofacial computed tomography (CT) performed 2 years earlier showing both TMJs in coronal view (**A**), right TMJ in sagittal view (**B**) and left TMJ in sagittal view (**C**)

During the medical history, the mother of the patient reported an otomastoiditis and periorbital cellulitis at the age of six recovered after antibiotic treatment with complete remission of symptoms. A maxillofacial computed tomography (CT) performed 2 years earlier showed condylar symmetry, normal dental formula and absence of significant radiographic alterations (Fig. 1A-C). The patient was referred to maxillofacial surgeon and dental specialist.

The maxillofacial surgeon reported a condition of inflammation with functional limitation during mouth opening and prescribed paracetamol and antibiotic (amoxicillin/clavulanic acid) assumption .

**Fig. 2:**
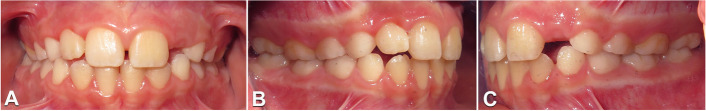
Intraoral photographs in frontal (**A**), right lateral (**B**) and left lateral (**C**) views

**Fig. 3:**
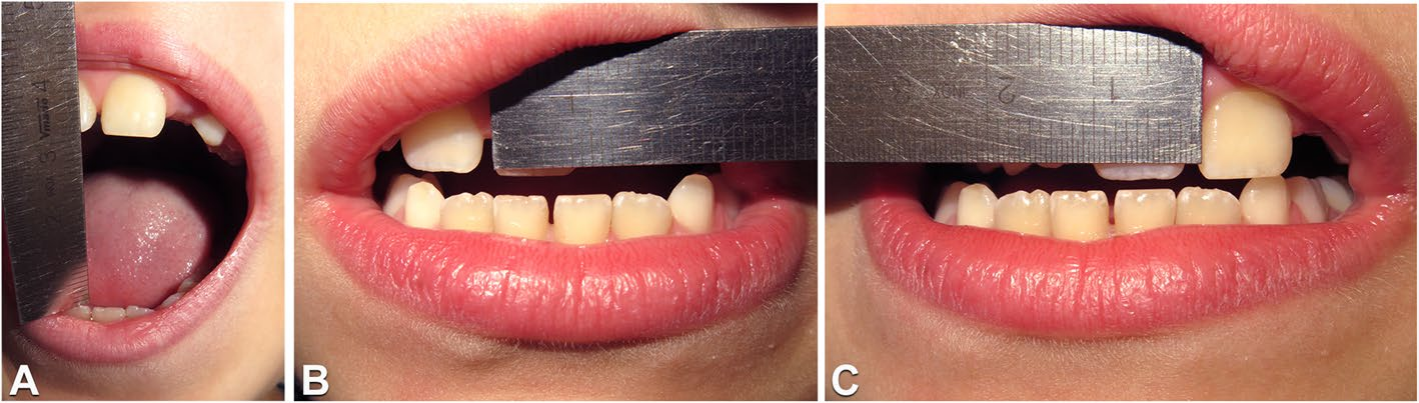
Evaluation of dynamic mandibular movements by mean of ruler: mouth opening measures 31 mm (**A**), left laterotrusion measures 6 mm (**B**), right laterotrusion measures 9 mm (**C**)

**Fig. 4:**
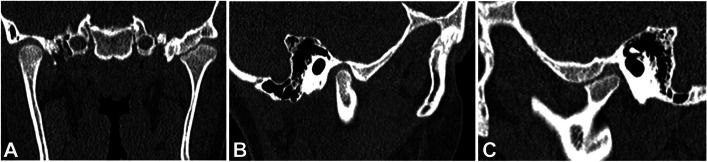
Images of maxillofacial computed tomography (CT) showing both TMJs in coronal view (**A**), right TMJ in sagittal view (**B**) and left TMJ in sagittal view which showed the mandibular condyle significantly erosed (**C**)

At the Dentistry Unit, the patient had complained a pulsating and intermittent pain (Visual Analogic Scale = 9) on the left side located in the preauricular area, at the gonial angle region and in the sub mandibular area for about 4 months. Further investigations included the DC/TMD symptom questionnaire and clinical examination [12, 13]. At the extra oral clinical evaluation, there is no evidence of significant facial asymmetry on the frontal view and the facial profile was orthognathic. At the clinical intraoral examination the patient presented a Class I malocclusion in mixed dentition, lower dental midline shift to the right and absence of any other relevant alteration. (Fig. 2A-C). Dynamic evaluation of the mandibular function showed reduced mouth opening (31 mm) with left uncorrected deviation of the mandible during mouth opening (Fig. 3A) and protrusion, limited right laterotrusion (6 mm) (Fig. 3B) compared to the left one (9 mm) (Fig. 3C). The clinical examination led to the diagnosis of bilateral myalgia and arthralgia of the left temporomandibular joint according to the DC/TMD [11, 12]. A maxillofacial CT showed the left condyle significantly eroded (Fig. 4C) when compared to the previous CT scan. The patient received counselling and instructions about physiotherapy [14–16] and pain management continued by administration of paracetamol as needed.

Differential diagnosis included onset of juvenile idiopathic arthritis, idiopathic condylar resorption, osteoarthritis, osteonecrosis. In order to perform a complete diagnosis, the patient was referred to the rheumatologist who performed blood test and Magnetic Resonance Imaging (MRI) of both temporomandibular joints. This last confirmed morphological alteration of the left condyle and the glenoid fossa with flattening of the articular eminence, presence of mild inflammation and reduced thickness of the disk that was still correctly in place.

**Fig. 5:**
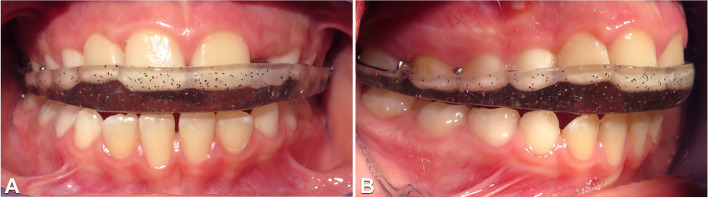
Intraoral photographs in frontal (**A**) and right lateral (**B**) views showing rigid bite plate

**Fig. 6:**
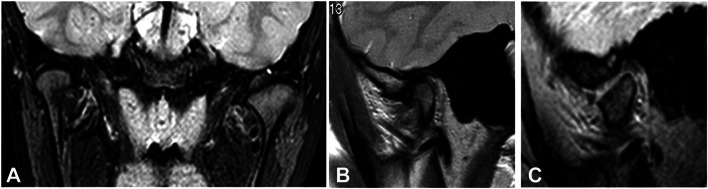
Images of TMJ MRI showing both TMJs in coronal view (**A**), right TMJ in sagittal view (**B**) and left TMJ in sagittal view(**C**)

**Fig. 7:**
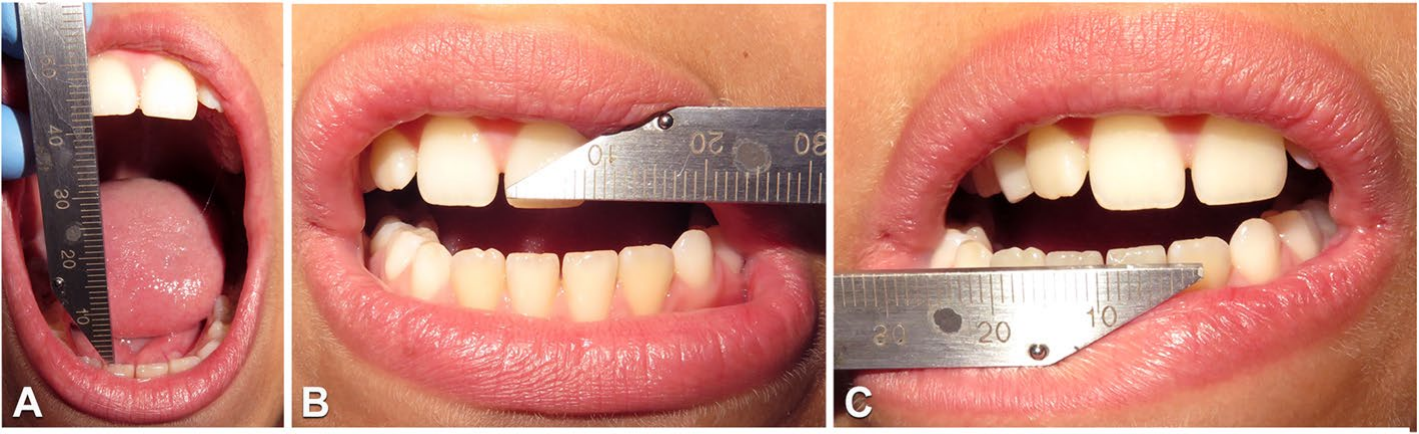
Evaluation of dynamic mandibular movements after therapy which shows an improvement in mouth opening (43 mm) (**A**), left laterotrusion measures 6 mm and right laterotrusion measures 9 mm

At the end of diagnostic path, the diagnosis was post-infective osteoarthritis of the left temporomandibular joint linked to the previous otomastoiditis. A customised passive rigid bite plate was realised, and bedtime use was prescribed (Fig. 5). One month later the full clinical examination according to DC/TMD revealed decrease of pain (Visual Analogic Scale = 4), persistence of arthralgia of the left temporomandibular joint, improvement of the mouth opening (40 mm), persistence of the left uncorrected deviation of the mandible during mouth opening and limited right laterotrusion. Three months later the patient reported improvement in the mandibular function and finally absence of pain (Visual Analogic Scale = 0). One year later the MRI showed absence of further alteration of the left condyle and the temporal bone (Fig. 6) and sensible reduction of the inflammatory infiltration on the left temporomandibular joint . During the last check up the patient showed an additional improvement of the mouth opening (43 mm) and the right and the left laterotrusion movements remain stable but without pain (Fig. 7). The follow up of the patient passed to 6 months in order to evaluate the need of orthodontic therapy closer to the pubertal spurt.

## Discussion and conclusions

We identified nine articles [3–7, 17–20] on TMJ septic arthritis of otogenic origin in children published since 2000 in English literature. Seven articles were case reports [3, 5, 7, 17–21] and they did not evidence any long-term consequences of septic arthritis of TMJ. On the other hand, Luscan et al. [3] observed in a prospective study of 45 patients that 1 patient had an erosion of the temporomandibular condyle and 2 patients presented clinical diagnosis of TMJ ankylosis as long-term complication, 4 and 16 months after otomastoiditis.

Burgess et al. [6] reported nine pediatric cases of otogenic septic arthritis of the temporomandibular joint in a retrospective study and showed that six patients presented a late ankylosis of TMJ from 0.5 to 4 years after the initial middle ear infection.

In this article, we present the management of a 9 years-old female patient with reduced mandibular movement, orofacial pain and condylar resorption that arose 2 years later an otomaoiditis.

The clinical presentation of our patient was characterised by many overlapping signs and symptoms making diagnosis difficult to perform at a glance. The patient had no history of trauma and the left otomastoiditis reported occurred about 2 years before the first symptoms of temporomandibular joint involvement. Differential diagnosis is crucial in such cases, where growing patients are involved, because the sequelae of temporomandibular disease may influence growth and mandibular function leading to severe facial asymmetry and permanent mandibular impairment [21, 22]. Dental practitioners need to be able to formulate differential diagnosis hypothesis and to address the patient to the right specialists. Moreover, conservative treatment, including counselling and physiotherapy, helps the patients in managing the acute situation with minimal risks preventing complications such as fibrosis and ankyloses of the temporomandibular joint [14, 15]. To date there is no consensus on treatment of post-infectious osteoarthritis [3]. Nevertheless, there is evidence for physiotherapy in being effective in most of TMD***s***, improving mobility of the temporomandibular joint and preventing adherences and ankyloses [14, 15]. The bone remodelling resulting from osteoarthritis is a permanent alteration of bone structure. However, condylar cartilage has itself a potential for bone modification and in growing patients there is as well a potential for condylar growth [23]. Modulation of bone remodelling and residual growth in growing patients [24] can be effective in improving facial symmetry by addressing the condylar asymmetry [21, 25]. In order to start any orthopaedic and orthodontic treatment, the inflammatory condition of the temporomandibular joint has to be under control and the mandible has normal function [21, 25, 26]. It is important to inform the patients and the families about these opportunities and to underline the importance of preserving mandibular function.

In our case, the patient improved mandibular function and full recovery of mouth opening without any pain during mandibular movement. Moreover, the conservative therapy and the use of a nocturnal bite plate helped pain management and were effective in reducing both myalgia and arthralgia of the left temporomandibular joint. The one-year control MRI showed stable bone morphology without progression of bone erosion and with no evidence of inflammation. The patient undergoes regular follow-ups. As soon as the pubertal growth spurt starts, we will consider the opportunity of undergoing orthopaedic treatment with a mandibular asymmetrical activator to address condylar asymmetry.

As previously suggested [6], this case highlights the importance of long-term follow-ups in children with acute media otitis or otomastoiditis due to TMJ disorders that can occur up to 4 years later.

Furthermore, in the multidisciplinary management of orofacial pain the role of the pediatric dentist is crucial for the diagnostic and therapeutic pathway to avoid serious impairment of mandibular function.

## Data Availability

Data sharing was not applicable to this case report because no datasets were generated or analysed during the study.

## ABBREVATIONS

CT: Computed Tomography
DC/TMD: Diagnostic Criteria of Temporomandibular Disorders
ENT: Ear nose and throat
MRI: Magnetic Resonance Imaging
TMD: Temporomandibular Disorders
TMJ: Temporomandibular joint
